# “Sentio ergo est”: Unmasking the psychological realities of emotional misperception

**DOI:** 10.1177/03010066241302996

**Published:** 2024-12-09

**Authors:** Myron Tsikandilakis, Persefoni Bali, Alexander Karlis, Patty Morfi, Pierre-Alexis Mével, Christopher Madan, Alison Milbank

**Affiliations:** School of Psychology, University of Nottingham, Nottingham, UK Medical School, Faculty of Medicine and Health Sciences, University of Nottingham, Nottingham, UK; School of Cultures, Languages and Area Studies, University of Nottingham, Nottingham, UK; School of Psychology, 6123 University of Nottingham, Nottingham, UK; Department of Physics, 68993 National and Kapodistrian University of Athens, Athens, Greece; School of Engineering, 6122 Nottingham Trent University, Nottingham, UK; School of Cultures, Languages and Area Studies, 6123 University of Nottingham, Nottingham, UK; School of Psychology, 6123 University of Nottingham, Nottingham, UK; Department of Philosophy, 6123 University of Nottingham, Nottingham, UK

**Keywords:** masking, consciousness, emotion, physiology, misperception

## Abstract

Perception is an important aspect of our personal lives, interpersonal interactions and professional activities and performance. A large body of psychological research has been dedicated to exploring how perception happens, whether and when it involves conscious awareness and what are the physiological correlates, such as skin-conductance and heart-rate responses, that occur when we perceive particularly emotional elicitors. A more recent and less explored question in psychological science is how and when misperception happens, and what are the physiological characteristics of the misperception of emotion. Therefore, in the current study, for the first time in relevant research, we recruited participants using trial-contour power calculations for false-positive responses, such as incorrectly reporting that a brief backward masked face was presented and thoroughly explored these responses. We reported that false-positive responses for backward masked emotional faces were characterised by pre-trial arousal, and post-trial arousal increases, high confidence ratings, and corresponding to stimulus-type misperception valence and arousal participant ratings. These outcomes were most pronounced for false-positive responses for fearful faces. Based on these findings, we discussed the possibility of a mechanism for partial self-encapsulated emotional-experiential apperception and the possibility of a fear primacy socio-emotional response module during combined visual ambiguity and high psychophysiological arousal.

A significant part of contemporary psychological research has been dedicated to exploring perception (Macmillan, 2002). This exploration involves whether perception can occur unconsciously and confer unconscious behavioural and emotional changes (Brooks et al., 2012). The idea of unconscious processing is currently subject to scientific debate (Goldstein & Young, 2022). As part of this debate, our group (Tsikandilakis et al., 2019a) and other researchers (Phillips, 2018) have acknowledged that the scientific pursuit for discovering whether there are unconscious responses is important. Nevertheless, while engaged in this important pursuit, we have neglected to explore a related critical question: Whether there are and what are the emotional responses that we experience when consciousness is wrong (Bennett, 2022)?

To answer this question, in this manuscript, we firstly elaborate on corrections and advances, and subsequently reverse-engineer methods used to explore unconscious responses. We use backward masking, signal detection theory (SDT), and SDT item null hypothesis testing and Bayesian analyses. We repurpose these methods and we provide an empirical report on a largely unaddressed but critical question in contemporary psychology: What do we emotionally experience when we consciously misperceive an emotional elicitor?

To experientially confront this critical question, we first need to appropriately address key aspects in relevant research. For example, backward masking is a method for visual suppression (Axelrod et al., 2015; Breitmeyer & Ogmen, 2000; Mulckhuyse & Theeuwes, 2010; Tsikandilakis et al., 2019a). Typically, it involves the brief presentation of a target image, such as a fearful face or an emotional scenery (Tsikandilakis et al., 2018; van der Ploeg et al., 2017). This is followed by a mask stimulus that is suggested to render the target imperceptible (Brooks et al., 2012; Gambarota et al., 2022; Meneguzzo et al., 2014; Mertens & Engelhard, 2020; van der Ploeg et al., 2017; see also Appendix 5). After the presentation, psychophysiological response assessments, such as subcutaneous sweating (SCR) and heart-rate (HR) and self-report rating responses (Bond et al., 2021; Haralabopoulos et al., 2020; Leong et al., 2023; Yu et al., 2022) are measured (Lo & Zeki, 2014; Minkin et al., 2019).

Relatedly, SDT is a method for measuring perceptual performance (Hautus et al., 2021). It involves three assessments: Sensitivity, specificity and accuracy (Macmillan, 2002; Verghese, 2001; Zhu et al., 2010). These assessments are based on the ratio of correct and erroneous responses for the perception of signal and noise (Abdi, 2007). The components that comprise signal detection assessments are hit and miss responses. Hit responses refer to correct responses. These include correctly discriminating targets from non-targets, called true positives (TP). These also include correctly discriminating non-targets from targets, called true negatives (TN). Miss responses refer to incorrect responses. These include erroneously responding that non-targets were targets, called false positives (FP). These also include erroneously responding that targets were non-targets, called false negatives (FN) (for a review, see Macmillan, 2002).

Backward masking can and should involve SDT metrics (McNicol, 2005; Smith & Ratcliff, 2009; Tsikandilakis et al., 2023b, pp. 3–7; Tsikandilakis, 2024, pp. 8–13). The purpose of backward masking is to render stimuli imperceptible or—as many researchers claim—unconscious (for dedicated reviews, see Bishop, 2008; Breitmeyer, 2015; Kouider & Faivre, 2017; Pessoa, 2005). The definition for responses to post-trial tasks for the detection or discrimination of masked stimuli that were imperceptible is that participants should respond by chance (Meyen et al., 2022), or in analogy to real-life as a blind person would (Erdelyi, 2004; Goldstein & Young, 2022).

As an indicative example, Pessoa et al. (2006) presented participants with masked fearful, happy and neutral faces for 33.33 and 66.67 ms with backward masking to neutral faces for either 83.33 or 111.67 ms. After each presentation, they asked the participants “Did you see a fearful face?” (Y/N). They showed that when using a percentage assessment, the participants’ responses were not significantly different to chance for 33.33 ms. When they assessed the responses using signal detection metrics, perceptual performance at 33.33 ms was significantly above chance. The researchers also identified a subgroup of participants who were significantly above average performance for recognising target stimuli at 33.33 ms, referred to as “overachievers.” They identified another group of people who were below chance-level performance for recognising target stimuli at 66.67 ms, referred to as “underachievers.”

These findings were replicated widely, and subsequent replications added that not only different individuals, but also different individuals for different stimulus types varied significantly for signal detection responses (Herzog et al., 2020; Mudrik et al., 2011; Phillips, 2016, 2018; Stein & Peelen, 2021; Zhang et al., 2012). The majority of these findings related to higher signal detection performance for fearful elicitors, such as fearful faces (Liddell et al., 2005). This was suggested to occur due to their evolutionary value. Relevant findings from the area of neuroscience suggested that fear occupies a dedicated subcortical pathway to arousal eliciting brain regions (Bang & Rahnev, 2017; Bishop, 2008; Carlson et al., 2009; Li et al., 2010; Mattavelli et al., 2021; Öhman, 2005; Öhman et al., 2007; Schlumpf et al., 2013; Sherman et al., 2015; Silverstein et al., 2015; VanRullen, 2011).

These findings, in addition to their evolutionary and neuroscientific importance (Modirrousta-Galian & Higham, 2022), illustrated that SDT metrics were the better choice compared to hit rates. They offered a quantum of compensation to researchers that for no less than the past 70 years had discoursed the biases of percentage metrics and discussed the potential benefits of SDT metrics for the assessment of perceptual performance during visual suppression (Banks, 1970; Lann, 1959; Macmillan, 2002; Pollack & Norman, 1964).

The aforementioned acknowledgement suggested that SDT items, such as FN, FP, TP and TN, can be used for the analyses of participants’ responses to backward masked stimuli. A state-of-the-art analysis in backward masking included the assessment of the psychophysiological responses to backward masked stimuli per SDT item. Psychophysiological outcomes were assessed between hits, such as typically TP responses, and miss responses, such as typically FN responses (Pessoa, 2005, pp. 368–371). Using these analyses, if a researcher could report outcomes for brain activation or peripheral nervous system arousal for FN reports to a target stimulus, they could claim that a participant was unconsciously influenced by a target stimulus. Conversely, if psychophysiological and self-report changes occurred only for TP responses, a researcher could claim that conscious awareness was involved in the participants’ responses (Adolphs, 2008; Pessoa & Adolphs, 2010; Winkielman & Schooler, 2012).

As an example, if we return to Pessoa et al. (2006), the researchers showed that fearful faces compared to neutral faces presented for 33.33 ms resulted in activation in the left and, more so, to the right amygdala, a central nervous system locus associated with the processing of fear (Adolphs et al., 1995; Davis, 1992, 1997; LeDoux, 2003; Öhman, 2005). They also reported activation in the right fusiform gyrus, a locus associated with face processing (McCarthy et al., 1997), expertise, and repeated exposure to an elicitor type (Gauthier et al., 1999), such as human faces (Weiner & Zilles, 2016). These findings were recorded for hits (TP) but not for miss responses (FN) for fearful faces (Pessoa et al., 2006, pp. 367–368). FN responses for fearful faces did not provide any evidence for lateral or bilateral activation of the amygdala and the right fusiform gyrus compared to any other presented stimulus type (Pessoa et al., 2006, pp. 369–374). These outcomes had a near paradigm-shift impact. They were replicated several times using fMRI, fNIRS, and EEG (Balconi et al., 2017; Brooks et al., 2012; Gambarota et al., 2022; Meneguzzo et al., 2014; Mertens & Engelhard, 2020; Rohaut & Naccache, 2017), SCR and HR (Beissner et al., 2013; van der Ploeg et al., 2017), and self-report rating responses (Bargh, 2013; Overgaard & Sandberg, 2021; Overgaard & Timmermans, 2010). They made a very strong case that “amygdala responses are modulated by awareness” (Pessoa et al., 2006, p. 369).

SDT-item analyses made a very strong argument: FNs do not confer psychophysiological and self-report rating emotional responses, TPs do. Awareness modulates our responses to emotional stimuli. Naturally, these findings also generated a very important subsequent question (Bargh, 2017; Bargh & Hassin, 2021): If awareness modulates our responses to emotion, what happens when our awareness is wrong (Koch et al., 2016)? Are there quantifiable emotional correlates that can show that we can experience something that was not there to be perceived, such as emotional responses to FPs for the perception of an emotional elicitor (Alexandrov & Sams, 2005; Barrett et al., 2007a, 2007b; Tracy et al., 2007; Tsuchiya & Adolphs, 2007)?

This question can be answered by repurposing our understanding of backward masking and signal-detection-item analyses. For example, research on backward masking up to 2017 involved only static durations for the presentation of a masked target (van der Ploeg et al., 2017; Wiens, 2006). With a mind that previous research had provided evidence that different participants (Kouider & Dehaene, 2007) and different stimulus types (Hedger et al., 2016) required different presentation durations for attaining chance-level perceptual performance, our group compiled a series of publications using individually adjusted thresholds for the presentation of backward masked stimuli (Tsikandilakis et al., 2018, 2019b, 2019c, 2020a, 2021a; Tsikandilakis & Chapman, 2018).

We invited participants to a session in which they were presented with pattern-masked facial stimuli, such as fearful, happy, angry, sad and neutral faces, and an equal number of pattern-masked Gaussian blurs we used sensitivity index A to measure their perceptual performance (Zhang & Mueller, 2005; for a dedicated section on SDT metrics, see *Main Experiment: Results: Analytical Framework*). We also used Bayesian analyses to explore if the data can be observed under the null hypothesis that participants’ performance was statistically proximate to chance (Dienes, 2014, 2015, 2016, 2019, 2021; for a dedicated section on Bayesian inference, see *Main Experiment: Results: Analytical Framework*).

In a second session, we presented the same participants with the same pattern-masked facial stimulus types as session one for the durations that previously provided Bayesian evidence for chance-level performance per participant and stimulus type. We assessed their physiology and self-report ratings. We showed strong Bayesian evidence for null responses for overall, hit (TP and TN) and miss (FP and FN) responses for all stimulus types for SCR, HR, and digital camera technology facial–emotional recognition analyses (Skiendziel et al., 2019), and participant self-reports (Meyen et al., 2022).

This method has been discoursed extensively in previous review papers (Tsikandilakis, 2024; Tsikandilakis et al., 2019a, 2020a, 2023b, 2023c, 2021a, 2022). It provided a solution to achieving individually adjusted unbiased chance level perception during backward masked (Stein & Peelen, 2021). Most critically, it provided us with empirical initiatives for exploring the emotional correlates of FP responses under conditions of backward masking (Gambarota et al., 2022). To explore whether our method of unbiased individually adjusted unconsciousness was not due to non-responder effects, such as the assessment of participants that did not respond with physiological arousal to emotional faces (Ellena et al., 2020; Skoluda et al., 2015; van der Ploeg et al., 2017), we implemented control conditions. In one condition, we explored using SCR, HR and facial–emotional recognition, and self-reports whether biased brief durations for masked fearful or happy or angry or sad or neutral faces, and image-blurs, could elicit emotional responses. An important exploratory objective for these controls was to record what—if anything—happens during the misperception of emotion.

We repeatedly found trends for higher physiological responses for FP for masked fearful, angry and happy faces and, therefore, potential evidence that by increasing the trial-contour and statistical power of an experiment, we could explore whether there was evidence for increased physiological arousal for FP responses. These outcomes made the case that we could take the exploration of misperception outside the purpose-specific features of our multiplex individualised-thresholds paradigm, and explore using backward masking the psychophysiological and self-report rating responses of what happens when we misperceive emotional elicitors, such as FP responses for emotional faces.

Therefore, in the current study, we explored the correlates of the misperception of emotional faces outside our individual-thresholds paradigm (Tsikandilakis et al., 2023b). For the first time in relevant research, we recruited the required *n* (the number of participants; Faul et al., 2009) for the expected *k* (the number of expected FP trial contour; Baker et al., 2021) to explore the psychophysiology of FP responses to emotional faces using a static durations paradigm for backward masking (Fabrigar et al., 2020; Flake et al., 2022; Ludwig, 2023; Shrout & Rodgers, 2018).

## Main Experiment

### Methods

*Aims and hypotheses:* In the current study, we presented participants with masked fearful, angry, happy, sad and neutral faces and image-blurs and measured the emotional correlates of each SDT response item (TP, TN, FP, and FN), with an emphasis on FP responses for these stimuli. The exploratory hypotheses of the current study were that FP responses for perception of an arousing stimulus type would involve higher physiological and emotional rating characteristics, and that these physiological and emotional rating characteristics could be more pronounced for fearful faces.

*Participants:* From the five most recent meta-analyses in backward masking (Brooks et al., 2012; Gambarota et al., 2022; Meneguzzo et al., 2014; Mertens & Engelhard, 2020; van der Ploeg et al., 2017), when studies used d′, A′ and A″ (*k* = 14) instead of hit rates (Tsikandilakis, 2024; Tsikandilakis et al., 2023b), and involved the same masked stimulus type duration (i.e., 16.67 ms) and stimulus types as the current study (fear, anger, sadness, happy and neutral), the participants’ signal detection performance was transformed into non-parametric index A (Zhang & Mueller, 2005, pp. 207–208; for the coding script, see https://osf.io/h2u9s). Mean uncorrected sensitivity $\left(\frac{A}{Stimulus-type\;responses}\right)\;$ (Düvel & Kopiez, 2022; see also https://www.cns.nyu.edu/∼david/handouts/sdt/sdt.html) in these studies was .625 (SD = .071).^1^ An a-priori power analysis conducted in G-power (Faul et al., 2009; see also https://clincalc.com/stats/samplesize.aspx) with trial contour sequence estimations (Baker et al., 2021; see https://shiny.york.ac.uk/powercontours/), showed that for the number of trials in the current experiment (k(6)_overall _= 200; see *Main Experiment: Results: Psychophysiological Framework and Analyses*; see Cacioppo et al., 2007, pp. 163–168),193 participants would be required for P_(1− β) _≥ .9 (η^2^_p _≥ .01; f ≥ .1; d ≥ .2; *p* ≤ .05; P (H1) ≥ .9; B > 3) (Giner-Sorolla et al., 2019, pp. 6–11; Kelter, 2021, pp. 5–14; Dienes, 2021, pp. 4–17).

A total of 197 participants (98 females) volunteered to participate in a preliminary screening stage. The exclusion criteria for the population sample were not being diagnosed or having been previously diagnosed with a DSM Axis I or II disorder, and current or previous alcohol/drug abuse through self-reports. The participants were also assessed with questionnaires. The participants were screened with the Emotional Reactivity Scale (ERC; Nock et al., 2008). The participants were also assessed for eligibility for being included using the Personality Disorder Questionnaire (PDQ; American Psychiatric Association, 2022), a modified version of the Moral Injury Questionnaire (MIQ; Koenig et al., 2019), the Somatic and Psychological Health Report Questionnaire (SPHRQ; Hickie et al., 2001), and an online multiple-trait component Alexithymia/Emotional Blindness questionnaire (Alexithymia, 2023). These thorough assessments were employed so that the participants’ self-reports could be validated (for a full review on this subject, see Tsikandilakis et al., 2023b, pp. 4–7; Tsikandilakis, 2024, pp. 9–11)

Data from two participants were excluded from further analysis due to having a SPHRQ score (>3) that indicated a possible psychiatric diagnosis. Data from a single participant were excluded from further analysis due to PDQ scores (>.5) that indicated a possible personality disorder. Data from a single participant were excluded from further analysis due to Alexithymia scores that indicated Alexithymic traits (≥94). The final population sample included 193 participants (97 females) with mean age 27.82 (SD = 3.11) years. The experiment was approved by the Ethics Committee of the School of Psychology of the University of Nottingham.

**Figure 1.**
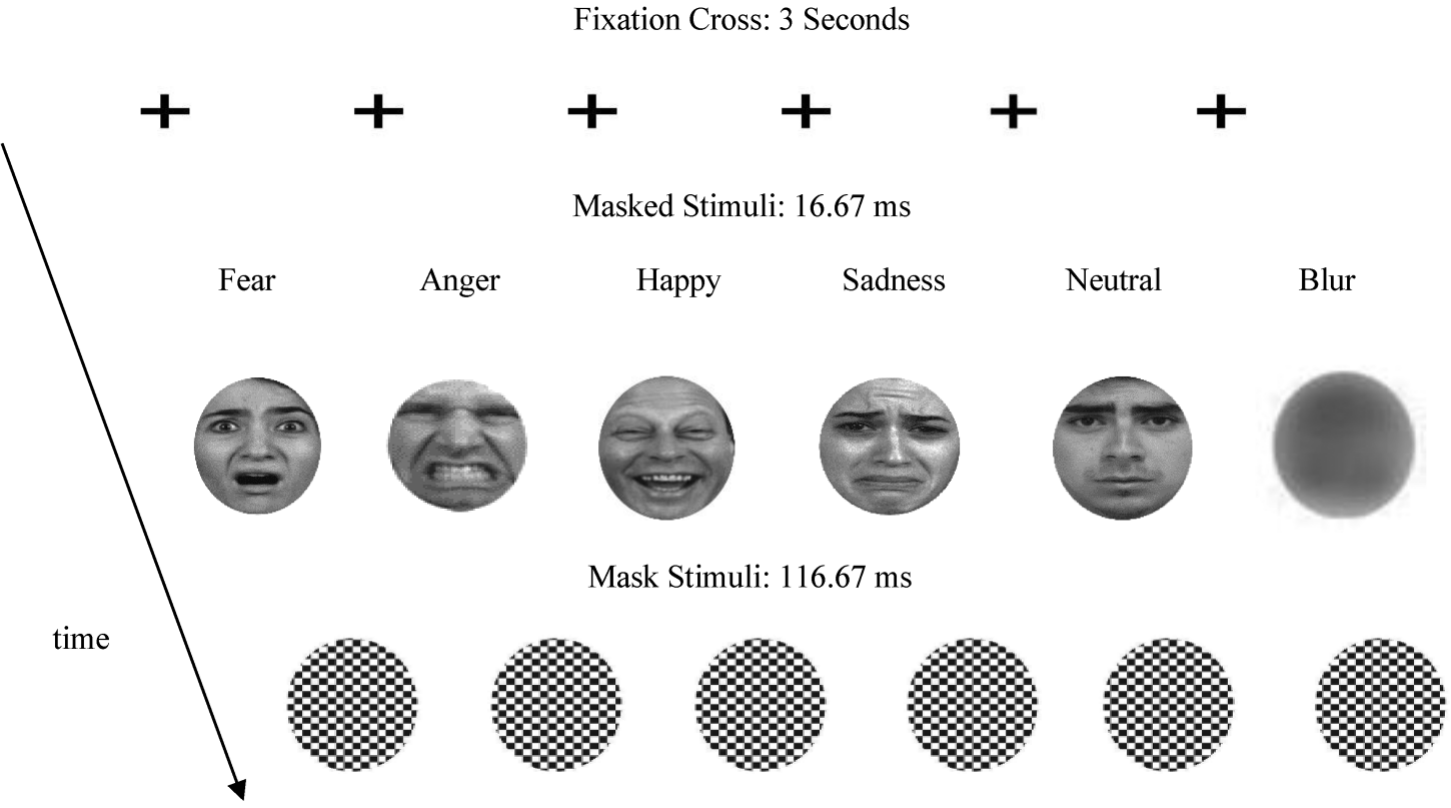
Types of faces (fearful, angry happy, sad, neutral and image blurs) presented during the main experiments (k_facial−stimulus type _= 20; k_blurs _= 100). The presentation was preceded by rating signal detection, discrimination and confidence Likert scales as well as ratings for valence and intensity, and combined physiological assessments as described in-text and elaborated in Tsikandilakis (2024) and Tsikandilakis and colleagues (2023b).

*Stimuli:* The facial stimuli used were taken from the dataset created by Gur and colleagues (2002). A total of 100 actors expressing fearful or angry or happy or sad or neutral expressions were used. The non-facial masked blurs were generated using pseudo-randomised pixel permutation in MATLAB (Tsikandilakis et al., 2021a, 2022). All stimuli included in the current experiment were selected based on that they had provided Bayesian evidence for equivalence of significance (Dienes, 2016) for within-category and stimulus-type SCR and HR responses and valence and intensity ratings (Sutton et al., 2019), and additional facial characteristics, such as attractiveness, and racial and cultural familiarity (Garrido & Prada, 2017), in previous studies by the current group (Tsikandilakis et al., 2018, 2019b, 2019c, 2019a, 2020a; Tsikandilakis & Chapman, 2018; see Appendix 1). The black-and-white mask patterns were created in MATLAB using random chess-board-box permutation using the same coding script that was applied in previous studies by the current group (Figure 1). All stimuli were adjusted for interpupillary distance, transformed to grey scale and resized to a 1024 × 768 pixels resolution. Their luminance was averaged using SHINE, MATLAB Toolbox, and they were spatially aligned and placed in a white circle (height: 6 cm, width: 4 cm; Mask _tile−size periphery _= .048 cm) (for a full description of rationale and procedures, see Tsikandilakis et al., 2023b, pp. 5–7; Tsikandilakis, 2024, pp. 18–23).

*Facial recognition Software:* Computer-based analysis of the participants was conducted with Noldus Face Reader 9.1 using an HD camera mounted at the bottom of the presenting screen and centred on the participants’ face. The analysis employed the highest video capture frames per second allowed by the equipment (30 fps). The analysis was run using the Viola-Jones cascaded algorithm with a 500-point Euclidean transformation points to eliminate static identification variability for image quality, lighting, background variation and orientation (Tsikandilakis, 2024). Each participant was evaluated as regards to an expressed emotion after controlling for the influence of action units that were present in their own neutral expressions using a participant calibration module (Tsikandilakis et al., 2019b, 2023a). The analysis included the in-built emotional categorisation labels in Noldus (anger, fear, surprise, happiness, sadness, disgust, and neutral). Facial–emotional recognition was defined as a categorical classification criterion (C_discrimination _≥ .6) for an emotional response up to 5 s post-stimuli offset (Cacioppo et al., 2007, pp. 267–273; Lewinski et al., 2014, pp. 7–14). Participants were aware that their facial expressions were recorded (van der Ploeg et al., 2017, pp. 141–143).

*Physiological assessment:* Combined physiological assessment was used as described in detail in our previous research (Tsikandilakis et al., 2018, 2020a, 2020b, 2020c, 2021a; Tsikandilakis & Chapman, 2018). Skin conductance responses (SCR) and heart rate (HR) were used. Skin conductance was measured from the left hand (index/first and middle/second fingers) of each participant using disposable Ag/AgCl gelled electrodes. The signals were received by a BIOPAC System, EDA100C in units of micro-siemens (μS) and recorded in AcqKnowledge (Braithwaite et al., 2013). The HR was measured via a single-finger sensor from the left hand (ring/third finger). The signal was measured by a BIOPAC System, PPG100C using infrared photoplethysmography of blood flow fluctuations and converted and recorded in beats per minute (bpm) in AcqKnowledge. The presence of a phasic SCR was defined as an unambiguous increase (μS ≥ .01) occurring up to three seconds post-stimuli offset (Cacioppo et al., 2007, pp. 163–167). The presence of a HR response was defined as an event-related HR peak in beats per minute occurring up to 5 s post-stimulus offset (Cacioppo et al., 2007, pp. 187–191). Each response was calculated using the inbuilt derive phasic from tonic and find cycles routines as the highest peak in physiological responses (δ) in respect to a tonic baseline averaged across the period (δT) of each pre-stimulus onset using parallel port-input derived onset markers (Braithwaite et al., 2013, pp. 38–41). For exploring pre-stimulus arousal, we measured the highest peak (δ_pre_) during a 3-s window before each stimulus onset in comparison to a non-concurring highest peak in physiological responses during either the first 3 s (SCR) or 5 s (HR, Noldus) of the post-trial sequence rest period (δ_post_). The signals were received by the same BIOPAC system, recorded in AcqKnowledge (Braithwaite et al., 2013), and compared for each signal detection response among different stimulus types (for a full review of this methodology, see Cacioppo et al., 2007, pp. 163–167).

*Procedures:* Participants were invited in a quiet laboratory space in the School of Psychology of the University of Nottingham*.* The participants completed the questionnaire assessments and were allowed a 5-min break before the main experiment. The main experiment included fearful or angry or happy or sad or neutral faces and an equal overall number of blur stimuli for 16.67 ms (*k*_face−type _= 20; *k*_blurs _= 100; and *k*_overall _= 200; see *Power Calculations*). Each stimulus was presented separately and masked with a 116.67 ms black-and-white pattern mask. The stimuli were presented with order randomised and number of actor-repetitions supervised by a manual Python array script (*k* = 2; see https://osf.io/xy9ru). The stimuli were validated for conferring strong Bayesian evidence (BF < .33; Dienes, 2014, 2015, 2016, 2019, 2021) for physiological and emotional rating characteristics in previous studies (Tsikandilakis et al., 2018, 2019c, 2020a, 2021a, 2023b; Tsikandilakis & Chapman, 2018). The stimuli were presented in an HD Lenovo monitor adjusted at 60 Hz (16.67 ms per frame) with dropped frames diagnostics; no instances of dropped frames reported (expected dropped frame rate = 1/5.000). The presentation was programmed in the coder and builder components of PsychoPy (Peirce, 2009).

The main experimental stage started with a 5-min on-screen training session during which participants were introduced to the terminology of the experiment and shown how to use the mouse or keyboard to respond to the engagement tasks (Ellemers et al., 2019; Gray et al., 2013; Mikels et al., 2005). An on-screen interval screen was then presented, and participants were asked whether they understood the instructions, and whether they were ready to proceed to the main experiment. The participants were given the choice to ask the attending researcher questions before they decided to proceed; no instances of required researcher feedback were reported. Each experimental trial started with an on-screen fixation cross for 3 s. This duration was chosen to allow for reliable pre-stimulus onset arousal assessments during FP trials (Cacioppo et al., 2007, pp. 163–167). After the fixation cross a single face or image blur was presented at fixation for 16.67 ms; order randomised. The image was immediately followed by a black-and-white pattern mask for 116.67 ms (Figure 1).

After the presentation, a blank screen was presented for 5 s. Participants were then asked to respond to a set of questions using the keyboard and mouse with order of questions randomised. They were asked whether they saw a face (Y/N) followed by a one (not at all) to nine (very) confident rating for their choice in a subsequent slide. Using conditional branching, if a participant replied “Yes,” they were presented with a list (fear, anger, happiness, sadness, neutral, other; order randomised) and asked, “What was the face expressing?.” If they replied “No,” they were presented with same list and asked, “What emotion better describes your experience during the presentation?.” Participants were asked to rate the intensity of the presented elicitor from one (very low) to nine (very intense). Participants were also asked to rate the valence of the presented elicitor from one (very negative) to nine (very positive). After the engagement tasks a 5-s blank screen interval was presented before the next trial. This duration was chosen for achieving reliable comparisons between pre-stimulus onset arousal and post-stimulus offset arousal assessments (Cacioppo et al., 2007, pp. 163–167, 187–191, 267–273). The main experiment lasted approximately 40–45 min. The overall length of the experiment was approximately 45–60 min.

### Results

*Analytical framework:* As a metric for signal detection performance, we used sensitivity index A based on advantages that A has compared to d′, A′, and A″. For example, compared to d′, A is a non-parametric index and does not include any assumptions concerning the shape of the underlying noise-to-signal distribution. Sensitivity index A can provide an index for zero values, such as zero hits or miss responses, and includes diagonal Euclidean corrections to the A′ and A″ metrics for scores that lie in the upper left quadrant of the ROC curve (i.e., False Alarms Rate ≤ 0.5 and Hit Rate ≥ 0.5; Zhang & Mueller, 2005, pp. 204–207). The analyses in every section were performed using null-hypothesis significance (NHST; Lakens et al., 2018) and Bayesian testing (Dienes, 2019). NHST was set at *p* ≤ .05 for omnibus ANOVA tests with full post-hoc comparisons without a-priori pairwise predictions (Wiggins & Christopherson, 2019) and including Bonferroni corrections ( $\frac{\alpha }{K}$ ) to avoid FP/Type-I and FN/Type II errors (Hollenbeck & Wright, 2017). Bayesian analyses followed the Dienes paradigm (Dienes, 2014, 2015, 2016). We defined evidence for the null hypothesis at a Bayes Factor (BF) < .33 given a non-contributing omnibus effect size of BF (H0) for Cohen's *f* = [0, ≤.05]. Evidence that the data could be observed under the alternate hypothesis were defined at B > 3 for a minimum effect size of interest of BF (H1) for *f* = [≥.1, +∞], and data that signify inconclusive results at .33 ≤ BF ≤ 3 at BF for f = [>.05, < .1] (Dienes, 2021). Instead of adjusting significance testing theory corrections to the reported BF values (Kruschke, 2010), evidence for post-hoc comparisons were defined based on minimum and maximum effects sizes of interest at BF (H0) for Cohen's *d* = [0, ≤ .1], BF (H1] for *d* = [≥.2, +∞] and inconclusive data at BF for *d* = [>1, < .2] (Dienes, 2023). Finally, like in previous publications, in order to present a clear as possible statistical account of the current items, we included, when mathematically applicable, three decimal points for each analysis (Tsikandilakis et al., 2022).

*Signal Detection Analyses:* To explore whether the trial power contour for this study was correctly calculated in relation to previous research (Mean A (SD) = .625 (.071); Brooks et al., 2012; Gambarota et al., 2022; Meneguzzo et al., 2014; Mertens & Engelhard, 2020; van der Ploeg et al., 2017) and assess the signal detection performance of the participants, we calculated sensitivity index A overall and for each stimulus type for detection performance. In the current study, non-parametric sensitivity index A for detection was overall not significantly different and provided strong Bayesian evidence for equivalence of significance to previous findings [Mean A (SD) = .626 (.059); *t*(192) = .455; *p* = .649; *d*_οne−sample _= .006 SE = .004; BF = .21; see Table 1; for one-sample *t*-test effect size calculations, see Tsikandilakis, 2024, pp. 11–12]. We conducted an additional analysis for subliminality (Erdelyi, 2004). NHST and Bayesian analyses showed that the current perceptual responses were statistically higher to absolute chance (.05) [*t*(192) = 29.983; *p* < .001; *d*_οne−sample _= 2.136; SE = .004; BF = + ∞; see also Table 1]. These results showed clearly that the presentation was not subliminal, and that participant and trial contour power calculations (Baker et al., 2021) enabled us to bring sensitivity between the current study and previous findings to very close proximity (Fleming, 2023; Goldstein & Young, 2022; Hausmann et al., 2021; Jessen & Grossmann, 2020; Lieberman, 2022).

**Table 1.** Perceptual characteristics per stimulus type.

	Sensitivity		Specificity
	Detection Mean (SD)	Discrimination Mean (SD)	Mean (SD)
Fear	.681 (.129)*	.191 (.02)*	.117 (.01)
Anger	.616 (.123)	.184 (.019)*	.133 (.009)
Happy	.629 (.131)	.183 (.018)*	.135 (.009)
Sad	.61 (.117)	.175 (.018)	.165 (.019)*
Neutral	.597 (.115)	.175 (.019)	.167 (.019)*
Blur			.169 (.02)*
Inferential ANOVA and Bayesian analyses Among stimulus types			
*p*	<.001	<.001	<.001
η^2^_p_ (f)	.068 (.27)	.11 (.35)	.68 (1.45)
BF (SE)	+ ∞ (.004)	+ ∞ (.001)	+ ∞ (.001)

*Note*. Mean (SD) Sensitivity for detection and discrimination, and specificity are presented and analysed per stimulus type. Mean (SD) non-facial blurs were presented when they contributed emotional perception context value (Specificity). Asterisks (*) show significance at Bonferroni-corrected *p* ≤ .01 values.

To further explore the perceptual performance of the participants, we assessed their sensitivity for discrimination. No previous data for comparisons with corrected discrimination SDT performance (Macmillan, 2002, pp. 44–52) were reported in previous studies (Brooks et al., 2012; Gambarota et al., 2022; Meneguzzo et al., 2014; Mertens & Engelhard, 2020; van der Ploeg et al., 2017). No comparisons could be made. We conducted an analysis for subliminality. NHST and Bayesian analyses showed that the current perceptual responses were statistically higher to corrected absolute chance defined as $A\;\left(\frac{\;1\;}{6}\right)=.167$ [Mean A (SD) = .178 (.019); *t*(192) = 7.384; *p* < .001; *d*_οne-sample _= .56; SE = .001; BF = + ∞; see Table 1]. These results showed clearly that the presentation for discrimination performance was not subliminal (Tsikandilakis et al., 2020a).

Further to these analyses, we explored perceptual specificity. We explored specificity for responses to the presented stimulus types (Atkinson, 1963; Corsello et al., 2007; Hautus et al., 2021; Macmillan, 2002; Swets, 2014; Swets & Green, 1978; Tsikandilakis, 2024; Tsikandilakis et al., 2019a, 2020a, 2021a, 2022, 2021b; Zwosta & Zenhausern, 1969). No previous data for comparisons with corrected specificity SDT performance were reported in previous studies. No comparisons could be made. We calculated the overall specificity and for each stimulus type. Overall corrected for multiple response-types specificity $\left(A\;\left(\frac{\;1\;}{6}\right)=.167\right)$ was A (SD) = .148 (.009), and was significantly lower than chance-level performance [*t*(192) = ‒42.251; *p* < .001; *d*_οne-sample _= 3.67; SE = .001; BF = + ∞; Table 1) suggesting that participants performed worse than chance at responding whether a target stimulus type was not presented (Hautus et al., 2021, pp. 297–303).

These calculations enabled us to provide NHST and Bayesian analyses for detection, discrimination and specificity per stimulus type for each signal detection metric (Pessoa, 2005; Pessoa et al., 2006, 2005). For detection [*F*(4, 768) = 13.938; *p* < .001; Mauchly's χ^2^ (9) = 11.319; *p* = .255; see Table 1], Bonferroni-corrected pairwise comparisons showed that fearful faces were higher for sensitivity for detection compared to angry (*p* < .001; *d* = .58; SE = .014; BF = + ∞), happy (*p* < .001; *d* = .417; SE = .013; BF = + ∞), sad (*p* < .001; *d* = .583; SE = .012; BF = + ∞) and neutral faces (*p* < .001; *d* = .692; SE = .012; BF = + ∞). No other significant pairwise comparisons were reported. These results showed that fearful faces were detected more accurately than any other presented facial stimulus type (Table 1).

For discrimination [*F*(4, 768) = 23.271; *p* < .001; Mauchly's χ^2^ (9) = 11.345; *p* = .253; see Table 1], Bonferroni-corrected pairwise comparisons showed that fearful faces were higher for sensitivity for discrimination compared to angry (*p* = .01; *d* = .389; SE = .002; BF = + ∞), happy (*p* = .003; *d* = .444; SE = .002; BF = + ∞), sad (*p* < .001; *d* = .889; SE = .001; BF = + ∞) and neutral faces (*p* < .001; *d* = .865; SE = .001; BF = + ∞). Angry faces were also higher than sad (*p* = .001; *d* = .556; SE = .002; BF = + ∞) and neutral faces (*p* = .001; *d* = .589; SE = .002; BF = + ∞). Happy faces were higher than sad (*p* = .001; *d* = .553; SE = .002; BF = + ∞) and neutral faces (*p* = .001; *d* = .555; SE = .002; BF = + ∞). Happy and angry faces were not significantly different and provided strong Bayesian evidence for equivalence of significance for discrimination [*t*(192) = .846; *p* = .399; *d* = .056; SE = .002; BF = .05]. These results showed that angry and happy faces were higher for discrimination to sad and neutral faces, and provided evidence for similar scores for discrimination performance, and fearful faces were discriminated more accurately than any other presented facial stimulus type (Table 1).

For specificity [*F*(3.93, 754.622) = 407.774; *p* < .001; Mauchly's χ^2^ (14) = 160.523; *p* < .001; ε = .79; Greenhouse–Geisser corrected; see Table 1], Bonferroni-corrected pairwise comparisons showed that incorrect responses for reporting seeing fearful faces were higher (i.e., lower specificity) compared to angry (*p* < .001; *d* = 1.62; SE = .001; BF = + ∞), happy (*p* < .001; *d* = 2.291; SE = .001; BF = + ∞), sad (*p* < .001; *d* = 4.812; SE = .002; BF = + ∞) and neutral faces (*p* < .001; *d* = 5.011; SE = .002; BF = + ∞), and non-facial blurs (*p* < .001; *d* = 5.031; SE = .002; BF = + ∞). Responses for angry faces were reported to be higher for misperception than sad (*p* = .001; *d* = 3.233; SE = .001; BF = + ∞) and neutral faces (*p* = .001; *d* = .3.411; SE = .001; BF = + ∞), and non-facial blurs (*p* = .001; *d* = .373; SE = .001; BF = + ∞). Responses for happy faces were higher for misperception than sad (*p* = .001; *d* = 2.72; SE = .001; BF = + ∞) and neutral faces (*p* = .001; *d* = 2.912; SE = .001; BF = + ∞), and non-facial blurs (*p* = .001; *d* = 3.191; SE = .001; BF = + ∞). Happy and angry faces were not significantly different for specificity [*t*(192) = 1.226; *p* = .222; *d* = .011; SE = .001; BF = .32]. Sad, neutral and non-facial blurs were significantly different [*F*(2, 384) = 3.194; *p* = .042; η^2^_p _= .016; SE = .002; BF = + ∞]. No Bonferroni-corrected pairwise comparisons survived further significance testing (*p* ≤ .01; BF > 3 (*d* ≥ .2); see Appendix 2). These results showed that incorrect responses for seeing angry and happy faces were high, and incorrect responses for seeing fearful faces were the highest among the current stimulus-type responses (Table 1).

*Psychophysiological Framework and Analyses:* To explore the psychophysiological correlates of FP responses in the current study, we adjusted and applied our previous analyses paradigm (Tsikandilakis et al., 2020a, pp. 7–11; Tsikandilakis et al., 2018, pp. 79–82; Tsikandilakis et al., 2019b, pp. 18–24). To present a clear illustration of the misperception of emotion under conditions of backward masking and avoid previous statistical-methodological confounds, the analyses were performed between each facial–stimulus type and non-facial blurs. This approach was undertaken to avoid confounds in previous—and potentially the current—research relating to responses that could erroneously result in double entries (McNeish & Wolf, 2020).

For example, previous research had not taken under consideration that for K _stimulus−types _> 2 an FP discrimination response for one facial–stimulus type (e.g., a fearful face) could also constitute a TP response for another facial–stimulus type (e.g., a sad face; or vice versa) resulting in double entries for a single response item (Macmillan, 2002, pp. 49–50). To avoid this hurdle, in the current analyses, TN and FN responses were drawn from each pool of facial stimuli, and TN and FP responses were drawn from the non-facial blur pool of stimuli separately for each stimulus type, providing single response items among facial stimulus types, while preserving the participant and trial contour of the initial parameters of the statistical power calculations for the current research [*k*(1)_blurs _= 100; *k*(6)_overall _= 200; P_(1− β) _≥ .9; η^2^_p _≥ .01; f ≥ .1; *d* ≥ .2; *p* ≤ .05; P(H1) ≥ .9; B > 3; Table 2Α and Β]. This method enabled a clearer analytical validity compared to previous approaches (Swets, 2014), as also previously illustrated by topical authors and the current research group (Kellen & Klauer, 2015; Macmillan, 2002; McNeish & Wolf, 2020; Pleskac & Busemeyer, 2010; Stanislaw & Todorov, 1999; Tsikandilakis et al., 2020a).

**Table 2.** Descriptive statistics, and effect sizes and BFs for SCR and HR.

Mean (SD)					
Α.		Pre-stimulus		Post-stimulus	
		SCR	HR	SCR	HR
Fear	TP	.006 (.003)	.993 (.114)	.075 (.013)*	5.44 (.778)*
	FP	.029 (.006)*	3.038 (.596)*	.072 (.017)*	5.346 (.787)*
	TN	.005 (.003)	1.017 (.127)	.011 (.003)	1.116 (.112)
	FN	.005 (.002)	1.011 (.117)	.01 (.004)	1.107 (.12)
Anger	TP	.006 (.003)	1.001 (.115)	.065 (.012)*	3.32 (.646)*
	FP	.019 (.006)*	2.747 (.407)*	.054 (.008)*	2.939 (.537)*
	TN	.006 (.003)	1.021 (.128)	.012 (.004)	1.11 (.118)
	FN	.004 (.003)	1.003 (.116)	.011 (.004)	1.08 (.11)
Happy	TP	.006 (.003)	.998 (.113)	.066 (.013)*	3.41 (.623)*
	FP	.019 (.005)*	2.778 (.402)*	.053 (.008)*	2.902 (.523)*
	TN	.005 (.003)	1.024 (.125)	.011 (.004)	1.107 (.119)
	FN	.004 (.003)	1.009 (.123)	.011 (.004)	1.126 (.121)
Sad	TP	.004 (.002)	.989 (.115)	.021 (.003)	1.099 (.113)
	FP	.006 (.003)	1.007 (.115)	.013 (.003)	1.112 (.12)
	TN	.006 (.003)	1.014 (.118)	.011 (.003)	1.108 (.119)
	FN	.004 (.003)	1.006 (.118)	.012 (.004)	1.109 (.116)
Neutral	TP	.006 (.003)	1.001 (.114)	.012 (.004)	1.1 (.108)
	FP	.005 (.003)	1.004 (.118)	.011 (.003)	1.101 (.12)
	TN	.004 (.003)	1.047 (.13)	.012 (.004)	1.109 (.119)
	FN	.004 (.002)	1.003 (.116)	.013 (.004)	1.111 (.122)
Standardised Cohen’s *d* (BFs)					
Β		Pre-stimulus		Post-stimulus	
		SCR	HR	SCR	HR
Fear	TP	.079 (.23)	.155 (1.37)	4.752 (+∞)*	5.993 (+∞)*
	FP	4.456 (+∞)*	4.354 (+∞)*	3.543 (+∞)*	5.772 (+∞)*
	TN	.133 (.97)	.123 (.86)	.097 (.29)	.111 (.76)
	FN	.154 (1.37)	.134 (.97)	.092 (.29)	.109 (.76)
Anger	TP	.074 (.23)	.146 (1.31)	4.018 (+∞)*	3.207 (+∞)*
	FP	2.383 (+∞)*	5.183 (+∞)*	3.83 (+∞)*	2.335 (+∞)*
	TN	.079 (.23)	.119 (.79)	.093 (.29)	.11 (.76)
	FN	.187 (2.71)	.143 (1.25)	.09 (.29)	.114 (.79)
Happy	TP	.081 (.23)	.149 (1.26)	3.865 (+∞)*	3.047 (+∞)*
	FP	2.727 (+∞)*	5.338 (+∞)*	3.69 (+∞)*	2.296 (+∞)*
	TN	.133 (.97)	.116 (.81)	.093 (.29)	.112 (.79)
	FN	.187 (2.71)	.134 (.97)	.092 (.29)	.106 (.76)
Sad	TP	.113 (.76)	.159 (1.37)	.18 (.2.71)	.111 (.78)
	FP	.074 (.23)	.137 (.97)	.097 (.29)	.108 (.76)
	TN	.075 (.23)	.126 (.89)	.094 (.29)	.109 (.76)
	FN	.187 (2.71)	.139 (.97)	.091 (.29)	.11 (.76)
Neutral	TP	.075 (.23)	.146 (1.31)	.093 (.29)	.112 (.79)
	FP	.013 (.09)	.141 (1.31)	.094 (.29)	.109 (.76)
	TN	.078 (.23)	.089 (.27)	.094 (.29)	.113 (.79)
	FN	.116 (.81)	.143 (1.41)	.095 (.29)	.108 (.76)

*Note*. In A, Mean and SD for SCR and HR. In B, standardised effect size Cohen's *d* (Fritz et al., 2012; Funder & Ozer, 2019) and standardised Bayes Factors (Dienes, 2019, 2021; Rouder et al., 2017) for SCR and HR. Asterisks (*) show statistical significance at *p* ≤ .001, and plus infinity (+∞) values for Bayesian analyses.

An analysis of variance with independent variables Type of Emotion (Fear, Anger, Happy, Sad and Neutral) and SDT-Item Response (TP, TN, FP and FN), and dependent variable pre-stimulus presentation SCR was conducted. The analyses revealed a significant effect of Type of Emotion [*F*(3.702, 710.697) = 490.306; *p* < .001; η^2^_p _= .719; Mauchly's χ^2^ (9) = 82.177; *p* < .001; ε = .768; Greenhouse–Geisser corrected; SE = .001; BF = +∞], a significant effect of SDT-Item Response [*F*(2.289, 439.416) = 2505.144; *p* < .001; η^2^_p _= _._929; Mauchly's χ^2^ (5) = 32.608; *p* < .001; ε = .941; Greenhouse–Geisser corrected; SE = .002; BF = +∞] and a significant interaction [*F*(8.015, 1538.828) = 427.653; *p* < .001; η^2^_p _= _._69; Mauchly's χ^2^ (77) = 447.913; *p* < .001; ε = .679; Greenhouse–Geisser corrected; SE = .001; BF = +∞]. All analyses showed violations of sphericity and were subjected to Greenhouse–Geisser corrections (Lane, 2016). These results showed that there were very large differences between each emotion, and signal detection item responses. Emphasis should be given to that the highest values for pre-stimulus presentations for SCR were for FP responses for fearful faces, followed by FP response for angry and happy faces, without significant differences for TP, TN, and FN among any stimulus-type combination (for full descriptive statistics and Bonferroni-corrected pairwise comparison, see Table 2Α and Β).

For pre-stimulus presentation HR responses an analysis of variance revealed a very significant effect of Type of Emotion [*F*(2.898, 556.435) = 1136.862; *p* < .001; η^2^_p _= .856; Mauchly's χ^2^ (9) = 517.379; *p* < .001; ε = .731; Greenhouse–Geisser corrected; SE = .004; BF = +∞], a significant effect of SDT-Item Response [*F*(1.525, 292.809) = 6405.837; *p* < .001; η^2^_p _= _._97; Mauchly's χ^2^ (5) = 293.021; *p* < .001; ε = .423; Greenhouse–Geisser corrected; SE = .006; BF = +∞] and a significant interaction [*F*(3.954, 759.21) = 1065.63; *p* < .001; η^2^_p _= _._847; Mauchly's χ^2^ (77) = 1532.77; *p* < .001; ε = .34; Greenhouse–Geisser corrected; SE = .009; and BF = +∞]. These results showed that the highest values for pre-stimulus presentations for HR responses were for FP responses for fearful faces, followed by FP responses for angry and happy faces, again without significant differences for TP, TN and FN among any stimulus-type combination (Table 2Α and Β).

For post-stimulus SCR, an analysis of variance revealed a significant effect of Type of Emotion [*F*(2.72, 530.398) = 3189.041; *p* < .001; η^2^_p _= .943; Mauchly's χ^2^ (9) = 227.948; *p* < .001; ε = .655; Greenhouse–Geisser corrected; SE = .001; BF = +∞], a significant effect of SDT-Item Response [*F*(2.168, 416.34) = 5059.548; *p* < .001; η^2^_p _= _._969; Mauchly's χ^2^ (5) = 159.321; *p* < .001; ε = .87; Greenhouse–Geisser corrected; SE = .001; BF = +∞] and a significant interaction [*F*(5.256, 1009.179) = 1139.748; *p* < .001; η^2^_p _= _._856; Mauchly's χ^2^ (77) = 1289.698; *p* < .001; ε = .44; Greenhouse–Geisser corrected; SE = .001; BF = +∞]. Emphasis should be given to that the highest values for post-stimulus presentations for SCR were for TP and FP responses for fearful faces, followed by TP and FP response for angry and happy faces, without significant differences for TN and FN among any stimulus-type combination (Table 2Α and Β).

For post-stimulus HR responses, an analysis of variance revealed a significant effect of Type of Emotion [*F*(2.842, 545.661) = 4425.506; *p* < .001; η^2^_p _= .958; Mauchly's χ^2^ (9) = 323.913; *p* < .001; ε = .716; Greenhouse–Geisser corrected; SE = .012; BF = +∞], a significant effect of SDT-Item Response [*F*(2.033, 390.242) = 6375.325; *p* < .001; η^2^_p _= _._971; Mauchly's χ^2^ (5) = 276.257; *p* < .001; ε = .675; Greenhouse–Geisser corrected; SE = .01; BF = +∞] and a significant interaction [*F*(4.889, 938.74) = 1494.051; *p* < .001; η^2^_p _= _._886; Mauchly's χ^2^ (77) = 2195.059; *p* < .001; ε = .413; Greenhouse–Geisser corrected; SE = .017; BF = +∞]. These results showed that the highest values for post-stimulus presentations for HR responses were for TP and FP responses for fearful faces, followed by TP and FP response for angry and happy faces, again without significant differences for TN and FN among any stimulus-type combination (Table 2Α and Β). The current results did not endorse that the reported physiological responses were due to subliminal processing (Tsikandilakis et al., 2020a). They endorsed the hypothesis that FP responses for arousing stimuli had high pre- and post-stimulus physiological correlates, particularly for fearful faces (Fisk & Haase, 2005; Pessoa, 2005; Pessoa et al., 2006).

*Facial–Emotional Recognition: Framework and Analyses:* For facial–emotional recognition, we used the same statistical framework described before. The analyses were performed for comparisons among facial types and SDT items. TP and FN responses were drawn from each pool of facial stimuli, and TN and FP responses were drawn from the non-facial blur pool of stimuli separately for each stimulus type. Emotional identification was based on criterion C, given a positive identification of a participants’ expression of an emotion set at C_discrimination _≥ .6 (Lewinski et al., 2014, pp. 7–14).

An analysis of variance with independent variables Type of Emotion (Fear, Anger, Happy, Sad and Neutral) and SDT-Item Response (TP, TN, FP and FN), and dependent variable pre-stimulus presentation emotional classification criterion C was conducted. The analyses revealed a significant effect of Type of Emotion [*F*(3.272, 628.171) = 5065.574; *p* < .001; η^2^_p _= .963; Mauchly's χ^2^ (5) = 213.248; *p* < .001; ε = .574; Greenhouse–Geisser corrected; SE = .003; BF = +∞], a significant effect of SDT-Item Response [*F*(1.715, 329.198) = 171.854; *p* < .001; η^2^_p _= .472; Mauchly's χ^2^ (9) = 68.904; *p* < .001; ε = .826; Greenhouse–Geisser corrected; SE = .003; BF = +∞] and a significant interaction [*F*(9.562, 1835.811) = 57.276; *p* < .001; η^2^_p _= .23; Mauchly's χ^2^ (77) = 300.145; *p* < .001; ε = .819; Greenhouse–Geisser corrected; SE = .004; BF = +∞]. These results showed that there were differences between each emotion, and signal-detection item responses. Emphasis should be given to that the highest values for pre-stimulus facial–emotional recognition were across all SDT-item response types for neutral faces, and also for FP for fearful faces only, although the latter did not reach the threshold of facial–emotional recognition (i.e., C_discrimination _≥ .6). No other significant differences were shown for TP, FP, TN, and FN among any stimulus-type combination (Table 3Α and Β).

**Table 3.** Descriptive statistics, and effect sizes and BFs for Noldus FaceReader.

Mean (SD)			
Α		Pre-stimulus	Post-stimulus
		Noldus FaceReader	Noldus FaceReader
Fear	TP	.426 (.055)	.67 (.121)*
	FP	.499 (.051)*	.665 (.122)*
	TN	.422 (.051)	.463 (.139)
	FN	.421 (.092)	.467 (.061)
Anger	TP	.429 (.054)	.657 (.124)*
	FP	.421 (.059)	.631 (.1)*
	TN	.428 (.053)	.451 (.137)
	FN	.427 (.093)	.469 (.063)
Happy	TP	.431 (.057)	.656 (.118)*
	FP	.421 (.058)	.618 (.101)*
	TN	.42 (.059)	.469 (.134)
	FN	.423 (.087)	.467 (.063)
Sad	TP	.427 (.057)	.66 (.127)*
	FP	.42 (.051)	.632 (.103)*
	TN	.421 (.051)	.464 (.131)
	FN	.427 (.091)	.461 (.064)
Neutral	TP	.723 (.059)*	.74 (.061)*
	FP	.715 (.058)*	.738 (.061)*
	TN	.723 (.055)*	.744 (.061)*
	FN	.722 (.063)*	.75 (.061)*
Standardised Cohen's *d* (BFs)			
Β		Pre-stimulus	Post-stimulus
		Noldus FaceReader	Noldus FaceReader
Fear	TP	.014 (.07)	1.725 (+∞)*
	FP	.623 (+∞)*	1.683 (+∞)*
	TN	.019 (.9)	.017 (.09)
	FN	.027 (.11)	.026 (.11)
Anger	TP	.039 (.14)	1.617 (+∞)*
	FP	.027 (.11)	1.4 (+∞)*
	TN	.031 (.12)	.107 (.23)
	FN	.023 (.09)	.043 (.14)
Happy	TP	.056 (.17)	1.608 (+∞)*
	FP	.027 (.11)	1.292 (+∞)*
	TN	.036 (.14)	.043 (.14)
	FN	.011 (.07)	.026 (.11)
Sad	TP	.02 (.09)	.043 (.14)
	FP	.036 (.14)	.026 (.11)
	TN	.027 (.11)	.014 (.07)
	FN	.002 (.03)	.024 (.09)
Neutral	TP	2.289 (+∞)*	2.308 (+∞)*
	FP	2.222 (+∞)*	2.209 (+∞)*
	TN	2.289 (+∞)*	2.342 (+∞)*
	FN	2.281 (+∞)*	2.392 (+∞)*

*Note*. In A, Winsorised Mean and SD values for Noldus FaceReader recognition criterion C (Pek & Flora, 2018). In B, standardised effect size Cohen's *d* and standardised Bayes Factors for Noldus FaceReader. Asterisks (*) show significance at *p* ≤ .001, and plus infinity values for Bayesian analyses.

For post-stimulus facial responses, an analysis of variance revealed a significant effect of Type of Emotion [*F*(3.59, 689.28) = 576.455; *p* < .001; η^2^_p _= .75; Mauchly's χ^2^ (9) = 58.049; *p* < .001; ε = .894; Greenhouse–Geisser corrected; SE = .004; BF = +∞], a significant effect of SDT-Item Response [*F*(2.661, 510.826) = 737.692; *p* < .001; η^2^_p _= .793; Mauchly's χ^2^ (5) = 58.048; *p* < .001; ε = .886; Greenhouse–Geisser corrected; SE = .003; BF = +∞], and a significant interaction [*F*(6.507, 1714.106) = 52.905; *p* < .001; η^2^_p _= .216; Mauchly's χ^2^ (77) = 436.514; *p* < .001; ε = .908; Greenhouse–Geisser corrected; SE = .007; BF = +∞]. Emphasis should be given to that the highest values for post-stimulus presentations for facial responses were for neutral faces for all SDT item responses. Additionally, TP and FP for fearful faces were higher compared to other emotion by response-type items, although, for all emotions TP and FP responses were above the C _discrimination _≥ .6 criterion. No other significant differences were shown for TN and FN responses among any stimulus-type combination (Table 3Α and Β). The current results did not show evidence that the facial–emotional responses were due to subliminal processing. They endorsed the hypothesis that FP responses for arousing stimuli had high facial–emotional recognition outcomes, particularly for fearful faces (Gross et al., 2000; Kring & Sloan, 2007; Kuppens et al., 2013).

*Rating Responses: Framework and Analyses:* For rating responses, we used the statistical framework described before for psychophysiology and facial–emotional responses. An analysis of variance with independent variables Type of Emotion (Fear, Anger, Happy, Sad and Neutral) and SDT-Item Response (TP, TN, FP and FN), and dependent variable Ratings for Valence was conducted. The analyses revealed a significant effect of Type of Emotion [*F*(3.727, 715.609) = 6183.855; *p* < .001; η^2^_p _= .97; Mauchly's χ^2^ (5) = 25.185; *p* = .003; ε = .916; SE = .017; BF = +∞], a significant effect of SDT-Item Response [*F*(2.725, 523.167) = 1103.479; *p* < .001; η^2^_p _= .852; Mauchly's χ^2^ (9) = 29.051; *p* < .001; ε = .916; Greenhouse–Geisser corrected; SE = .014; BF = +∞] and a significant interaction [*F*(8.319, 1597.245) = 2304.728; *p* < .001; η^2^_p _= .923; Mauchly's χ^2^ (77) = 654.237; *p* < .001; ε = .702; Greenhouse–Geisser corrected; SE = .027; BF = +∞]. These results showed that highest values for valence were for TP and FP responses for fearful faces, and TP and FP responses for angry and happy faces. The lowest values for valence were for TP and FP responses for sad faces. No other significant differences were shown for TN and FN among any stimulus-type combination (Table 4Α and Β).

**Table 4.** Descriptive statistics, and effect sizes and BFs for participant ratings.

Mean (SD)				
A		Valence	Intensity	Confidence (DSCR)
Fear	TP	2.356 (.163)*	7.808 (.188)*	8.178 (.348)*
	FP	2.417 (.211)*	7.746 (.146)*	7.902 (.542)*
	TN	5.114 (.33)	5.037 (.303)	6.346 (.36)
	FN	5.154 (.589)	5.022 (.288)	6.349 (.573)
Anger	TP	2.709 (.087)*	7 (.309)*	7.668 (.282)*
	FP	2.799 (.148)*	6.021 (.321)*	7.046 (.624)*
	TN	5.198 (.311)	5.045 (.32)*	6.259 (.373)
	FN	5.171 (.584)	5.021 (.281)	6.261 (.577)
Happy	TP	8.054 (.296)*	7.004 (.285)*	7.622 (.276)*
	FP	7.794 (.144)*	6.058 (.306)*	7.061 (.604)*
	TN	5.116 (.315)	5.061 (.308)	6.262 (.378)
	FN	5.126 (.621)	5.022 (.303)	6.263 (.629)
Sad	TP	3.132 (.338)*	3.01 (.312)*	6.267 (.278)
	FP	3.311 (.4)*	2.987 (.259)*	6.268 (.564)
	TN	5.138 (.507)	5.044 (.309)	6.263 (.36)
	FN	5.113 (.291)	5.039 (.454)	6.301 (.613)
Neutral	TP	5.193 (.561)	2.489 (.305)*	6.307 (.287)
	FP	5.194 (.576)	1.946 (.313)*	6.369 (.626)
	TN	5.166 (.487)	5.022 (.325)	6.318 (.371)
	FN	5.112 (.299)	5.031 (.463)	6.361 (.609)
Standardised Cohen's *d* (BFs)				
B		Valence	Intensity	Confidence (DSCR)
Fear	TP	7.752 (+∞)*	8.8 (+∞)*	3.939 (+∞)*
	FP	7.584. (+∞)*	8.601 (+∞)*	3.445 (+∞)*
	TN	.144 (1.17)	.139 (1.67)	.009 (.09)
	FN	.044 (.17)	.187 (2.71)	.003 (.03)
Anger	TP	6.779 (+∞)*	6.194 (+∞)*	2.804 (+∞)*
	FP	6.532 (+∞)*	3.035 (+∞)*	1.501 (+∞)*
	TN	.077 (.23)	.123 (1.21)	.196 (.2.89)
	FN	.003 (.03)	.19 (.2.87)	.192 (.287)
Happy	TP	7.945 (+∞)*	6.206 (+∞)*	2.74 (+∞)*
	FP	7.229 (+∞)*	3.155 (+∞)*	1.53 (+∞)*
	TN	.149 (1.28)	.108 (.91)	.189 (2.71)
	FN	.121 (.79)	.187 (2.71)	.188 (2.71)
Sad	TP	5.614 (+∞)*	6.677 (+∞)*	.179 (2.54)
	FP	5.121. (+∞)*	6.751 (+∞)*	.177 (2.53)
	TN	.088 (.27)	.116 (1.23)	.188 (2.71)
	FN	.157 (1.36)	.132 (1.54)	.106 (.54)
Neutral	TP	.063 (.19)	8.358 (+∞)*	.093 (.29)
	FP	.066 (.19)	10.109 (+∞)*	.041 (.14)
	TN	.011 (.09)	.187 (2.71)	.069 (.21)
	FN	.132 (.79)	.158 (.198)	.024 (.11)

*Note*. In A, Winsorised Mean and SD for participant ratings. In B, standardised effect size Cohen's *d* and standardised Bayes Factors for participant ratings. Asterisks (*) show significance at *p* ≤ .001, and plus infinity values for the Bayesian analyses.

For intensity, an analysis of variance revealed a significant effect of Type of Emotion [*F*(3.79, 727.659) = 10908.445; *p* < .001; η^2^_p _= .983; Mauchly's χ^2^ (9) = 22.927; *p* = .006; ε = .956; Greenhouse–Geisser corrected; SE = .011; BF = +∞], a significant effect of SDT-Item Response [*F*(3, 576) = 568.751; *p* < .001; η^2^_p _= .748; Mauchly's χ^2^ (5) = 1.567; *p* = .906; ε = .998; SE = .01; BF = +∞] and a significant interaction [*F*(10.204, 1959.155) = 4234.986; *p* < .001; η^2^_p _= .957; Mauchly's χ^2^ (77) = 204.714; *p* < .001; ε = .901; Greenhouse–Geisser corrected; SE = .022; BF = +∞]. These results showed that the highest values for intensity were TP and FP responses for fearful faces, and also for TP and FP response for angry and happy faces. The lowest responses for intensity were for TP and FP responses for sad and neutral faces. No other significant differences were shown for TN and FN responses among any stimulus-type combination (Table 4Α and Β).

For confidence for emotional discrimination, an analysis of variance revealed a significant effect of Type of Emotion [*F*(4, 768) = 426.039; *p* < .001; η^2^_p _= .689; Mauchly's χ^2^ (5) = 10.386; *p* = .32; ε = .986; SE = .017; BF = +∞], a significant effect of SDT-Item Response [*F*(2.557, 490.892) = 1135.727; *p* < .001; η^2^_p _= .855; Mauchly's χ^2^ (9) = 66.972; *p* < .001; ε = .859; Greenhouse–Geisser corrected; SE = .016; BF = +∞] and a significant interaction [*F*(9.857, 1892.592) = 148.913; *p* < .001; η^2^_p _= .437; Mauchly's χ^2^ (77) = 282.996; *p* < .001; ε = .844; Greenhouse–Geisser corrected; SE = .021; BF = +∞]. These results showed that the highest values for confidence were for TP and FP responses for fearful, and also for angry and happy faces. No other significant differences were shown for any other SDT-item responses among any stimulus-type combination (Table 4Α and Β). The current results did not show evidence for subliminal processing. They endorsed the hypothesis that FP responses for arousing stimuli would have distinguishable participant ratings, such as very high ratings for intensity for fearful, angry and happy faces (Sutton et al., 2019).

## Discussion

### Summary of Findings

In the current manuscript, we showed that the misperception of emotion involves emotional characteristics. We showed that the misperception of fearful, angry, happy, sad and in some respects even neutral faces is preceded by physiological arousal and stimulus-type specific physiological characteristics. It is also succeeded by physiological arousal and involves self-report ratings for valence and intensity that correspond to the misperceived emotion. Our findings suggest that the misperception of emotion is not a random residual or mere error, it is a meaningful emotional process that is influenced by the preceding physiological arousal that we experience before the perception of a condition and characterises what we experience and how we evaluate that condition after its perception.

### General Discussion

The first acknowledgement that should be submitted in the general discussion of the current manuscript is that we have provided thorough, clear and detailed empirical outcomes that the misperception of emotion can involve emotional characteristics. In this study, we used participant (Faul et al., 2009) and trial-contour power calculations (Baker et al., 2021), combined psychophysiological (Cacioppo et al., 2007) and self-report assessments (Craig et al., 2020), SDT metrics (Zhang & Mueller, 2005) and analyses (Pessoa, 2005), combined NHST (Lakens et al., 2018) and Bayesian analyses (Dienes, 2016), and applied advances in visual psychophysics (Breitmeyer, 2015; Breitmeyer & Ogmen, 2000; Yildirim et al., 2021), We demonstrated that emotion is involved in the misperception of masked faces expressing fear, anger, happiness, sadness and even neutral faces.

We showed that the misperception of fear was very common, and that it included high pre-stimulus-exposure and post-stimulus-exposure psychophysiological increases, and negative valence and high arousal ratings. We also showed that the misperception of anger and happiness involved pre-stimulus-exposure and post-stimulus-exposure increases in psychophysiology, and negative valence and high intensity ratings, and positive valence and high intensity ratings, respectively. The misperception of anger and happiness was less common and involved lesser psychophysiological and self-report rating changes compared to fear. The misperception of sad and neutral faces was the least common, and involved ratings for negative valence and low intensity, and average valence and low intensity, respectively.

These findings can be interpreted to suggest that our first emotional incitement when experiencing arousal under conditions of visual ambiguity in an emotionally plural ecological environment is because we assume a palpable cause for fear, possibly due to an unperceived source of potential threat (Dunsmoor et al., 2017). This experience can subsequently be attentionally re-directed upon an innocuous personal evaluation or interpersonal interaction (Szcześniak, 2023, 2024, Szcześniak & Řeřicha, 2024; Zebrowitz & Montepare, 2008). Concerning fear, in particular, the concept that fear can take attentional and experiential primacy over other—so called—basic emotions (Ekman & Cordaro, 2011) is common (Öhman, 2005) but it is not indisputable (Ortony, 2022; Storbeck & Clore, 2007). For example, researchers in a plethora of previous studies, have argued for the attentional primacy of anger and happiness due to their social communication value (Kristjánsson, 2010; Kristjánsson & Ásgeirsson, 2019; Lassalle & Itier, 2013). The current results were not consistent with these findings and supported that fear could take experiential and attentional primacy over anger and happiness, possibly because it confers evolutionary important survival value (Öhman et al., 2007). As follows, it is also worth mentioning that it is an issue of polemical debate whether this effect could be due to evolutionary survival value, involving a direct subcortical neural pathway from the visual thalamus to limbic structures, such as the amygdala (Liddell et al., 2005), or whether it is due to the high visual salience and high distinguishability of fearful elicitors (Gray et al., 2013; Morris et al., 1997; Pessoa et al., 2005), or, furthermore, synergistically, an epiphenomenal effect of fear-related evolutionary survival value via highly perceptible visual-emotional characteristics (Ney et al., 2022).

To the extent that subcortical thalamic access to the amygdala has been used as the conceptual progenitor for inferring subliminal fear responses (Brooks et al., 2012), we did not report any evidence for subliminal processing. On the contrary, FP responses in the current research provided non-significant differences and Bayesian evidence for equivalence for the null among all included stimulus types. Subliminal processing was not validated in any form in the current research. If, on the other hand, consciousness can be said to have been implicated in the current results (Bachmann & Aru, 2023), it was implicated via “error” (Frith, 2021). The participants did not respond in any way to faces that were presented but were imperceptible, they responded to faces that were not presented but were reported as being perceivable (Pessoa & Adolphs, 2010).

This is important in the sense that it showed that the misperception of emotion had peripheral nervous system and facial-expressive emotional characteristics. These were shown to precede and succeed the presentation of the non-arousing stimuli to which these emotional correlates were attributed. This is not a theoretical or speculative conjecture: It is for the first time in relevant research an illustrated empirical outcome (Brosch et al., 2007). False-positive responses for fear, anger and happiness involved pre-stimulus-exposure and post-stimulus-exposure psychophysiological arousal, and this effect was also involved in conscious reports for the evaluation of the valence and intensity of the misperceived stimuli. That could be interpreted to signify that pre-stimulus-exposure arousal was involved in the misperception of fear, happiness and anger. It influenced further automatic and involuntary post-stimulus-presentation responses, such as SCR, HR, and facial–emotional expressions. It also influenced conscious evaluations for self-reports.

These outcomes open a pathway to further empirical explorations of emotional misperception and raise a plethora of experimental possibilities. For example, we showed that with the exception of false-positive responses for fear, we were not able to show pre-stimulus-exposure facial–emotional changes for anger, happiness, and sadness, despite that FP reports for these stimuli had both pre-stimulus and post-stimulus-exposure SCR and HR, and self-report emotional correlates, respectively. That could be interpreted to signify that the experience and the expression of emotion have different emotional sensitivity thresholds (Metts & Planalp, 2011), and that the expression of emotion is potentially influenced by inhibition effects, such as self-presentation biases (Barrett et al., 2019). These could also signify that SCR and HR can be more discriminative psychophysiological markers for the experience of arousal compared to even currently state-of-the-art digital facial–emotional-recognition assessments (Marneweck et al., 2013).

Although we chose not to encumber our already plural analyses with non-significant outcomes, we did not report gender differences in any psychophysiological or self-report rating assessment throughout our current results (see Appendix 3; see also https://osf.io/9kdp6). Some researchers suggest that it should be an indisputably accepted empirical expectance that the female psychophysiology, due to higher amygdala sensitivity (Abbruzzese et al., 2019), should show higher peripheral nervous system emotional correlates in response to emotional elicitors (Bianchin & Angrilli, 2012). Our current results did not support this hypothesis. Currently, this effect might not necessarily be surprising given that the stimuli used in the present research were controlled in a multitude of previous studies for within-stimulus-category type and between and within gender differences for emotional recognition for signal detection and discrimination performance, psychophysiological assessments and several self-report assessments, such as intensity, valence, attractiveness, and racial and cultural familiarity (Tsikandilakis et al., 2019a, 2020a; Tsikandilakis & Chapman, 2018; Tsikandilakis et al., 2018, 2019b; see Appendix 1).

That can be interpreted to suggest that the internal validity of the current findings operated against the ecological validity of the current findings. For example, we have previously provided evidence that anger and hostility have different psychophysiological, facial–expressive and emotional–intentional characteristics, and that these effects included—among other contributing variables (Adler, 2014)—gender differences for the perception of anger and hostility (Tsikandilakis et al., 2020b). Conversely, and looking at the larger picture of emotional perception and misperception, we have demonstrated that sadness involves emotional subcategories with distinguishable psychophysiological characteristics and distinct eliciting circumstances, that is, melancholy, misery, bereavement and despair (Tsikandilakis et al., 2023a). Other research groups have claimed to have conceptually been able to separate the six basic emotions, that are commonly used in relevant research, i.e., fear, anger, happiness, sadness, surprise and disgust (Ekman & Cordaro, 2011; Wolf, 2015), to more than 100 distinguishable emotional states, including love, jealousy, admiration, trust, anticipation, guilt, contentment, calmness and rejection (Barrett, 2006). This raises pertinent issues and questions for topical research that are subject to contemporary and fierce argumentative debate (Frijda, 2017). Although the resolution of these issues is outside the scope of the current manuscript, it is worth considering whether we can be content with testing exclusively basic emotions, or more relevantly to the current research, very thoroughly controlled emotional stimuli, in empirical research relating to emotional perception and misperception (Hogenelst et al., 2015). More challengingly, it is worth considering whether there are truly indivisible basic emotions that should lead and label topical empirical research relating to emotional perception and misperception (for comprehensive reviews, see Barrett & Russell, 2014; Ortony, 2022).

Having stated these, and to reach a critical point in our discussion, the current outcomes can include further research possibilities and applications. These could relate to assessments for misperception in critical-care and critical-decision-making professions (McGrath et al., 2010). They could include the assessment and the understanding of interpersonal communication and miscommunication (Van Kleef, 2017). These could also inform the study of the phenomenology of self-assessment and self-awareness, and potentially the workings of human perceptual and conscious processes *in vivo* (Adler, 2014). To expand on these potentials in future research we must first recognise, communicate and make explicit an important distinction that stems from the current findings and related to the controversial notion of subliminal processing (Brooks et al., 2012) and also emotional misperception: Subliminality proposes a split between conscious awareness and emotional responses (Lucini et al., 2019). On the other hand, misperception—as demonstrated in the current research—shows synergy between consciousness and emotion. We have shown clearly and thoroughly that psychophysiological changes and conscious meta-cognition resonated and are both influenced by “ghost emotions,” such as the distinguishable and quantifiable characteristics of false-positive responses for emotional misperception (Block, 2007; Grof, 2019; Lambie & Marcel, 2002; Pekala, 2013).

Decisively, therefore, to address the philosophical intimations of the current manuscript, an important consideration—that is lacking in our contemporary psychological reasoning (Stein & Peelen, 2021)—is that conscious awareness and responses that have been labelled as unconscious, such as automatic and involuntary peripheral nervous system responses (Barrett et al., 2007), should not be reduced to merely separate modalities that can be divided to purely Cartesian dualisms (Pessoa & Adolphs, 2010). They can be parallel and corresponding processes forming a singular whole (Greenwald & Banaji, 2017) and we can frame this whole as subjective experience in tandem (Bargh & Morsella, 2008). When it comes to circumstances that involve emotional misperception, we can automatically and involuntarily think what we automatically and involuntarily feel; and vice-versa (Koch & Crick, 2001). The misperception of emotion can involve emotional characteristics (Bargh, 2013), and can involve thinking as we feel (Bargh, 2017), and feeling as we think (Smith & Lane, 2016). Therefore, these should be—as provocatively as our argument can be phrased—our own “Meditations on First Psychology” (see Descartes, 1641/2013): I feel therefore it exists; and not merely and singularly vice-versa.

### Limitations and Further Research

The current results are important. Nevertheless, they were tested using a specific experimental method: Backward masking (van der Ploeg et al., 2017; see Appendix 4). Future research could benefit from a replication of the current results with other visual suppression methods, such as binocular rivalry and continuous flash suppression (Tsuchiya et al., 2006). Further research could also benefit from a replication of the current results with methods, such as overt priming and cuing paradigms (Kiesel et al., 2010), that do not catalogue in experimental research directly relating to visual suppression (Francis, 2012; Kristjánsson & Ásgeirsson, 2019).

As discoursed in the *General Discussion* section of this manuscript, we have shown very strong evidence that the misperception of emotion is characterised by emotional correlates. We did not—and we did not intend to—address how the emotional correlates that precede or cause misperception occur. This is not an omission. The answer to this very critical question lies within the current data and relates, briefly, to the depletion of cognitive reserves for perceptual performance, conditional pre-trial preparation/anticipation effects and most intriguingly to default/constitutional emotional misinterpretation of noise arousal (Gerber & Green, 1999; Pronin, 2007). The discourse of these effects was not part of the scope of the current manuscript (see *Main Experiment: Aims and Hypotheses*). They require and were elected to be addressed in a dedicated manuscript (Tsikandilakis et al., 2024; see also McBride, 2023).

### Conclusions

In this manuscript, we showed that the misperception of emotion is not a meaningless response that is devoid of experiential content. We showed that the misperception of emotion can be emotional misperception to the extent that it shows preceding and succeeding changes in psychophysiology and emotional rating assessments. When we misperceive emotion, automatic and involuntary, albeit, not according to the current data subliminal, changes are involved in misperception. These changes impact and influence conscious self-reports for the emotional characteristics of a misperceived item.

## Notes

1. No studies that fit our criteria included per stimulus type participants’ signal detection metrics (Brooks et al., 2012; Gambarota et al., 2022; Meneguzzo et al., 2014; Mertens & Engelhard, 2020; van der Ploeg et al., 2017). The overall reported signal detection performance was used to provide trial-contour statistical power calculations (Baker et al., 2021).
